# Serum transfer RNA‐derived fragment tRF‐31‐79MP9P9NH57SD acts as a novel diagnostic biomarker for non‐small cell lung cancer

**DOI:** 10.1002/jcla.24492

**Published:** 2022-05-16

**Authors:** Jipeng Li, Chao Cao, Laifu Fang, Wanjun Yu

**Affiliations:** ^1^ Department of Central Laboratory, The Affiliated People's Hospital Ningbo University Ningbo China; ^2^ Department of Respiratory and Critical Medicine Ningbo First Hospital Ningbo China; ^3^ Department of Pathology, The Affiliated People's Hospital Ningbo University Ningbo China; ^4^ Department of Respiratory and Critical Medicine, The Affiliated People's Hospital Ningbo University Ningbo China

**Keywords:** biomarker, NSCLC, tRF‐31‐79MP9P9NH57SD, tRNA‐derived small RNAs

## Abstract

**Background:**

tRNA‐derived fragments (tRFs) have been found to have a crucial function in the pathophysiology of cancers. However, the function of tRFs in non‐small cell lung cancer (NSCLC) is yet unknown. The goal of this study was to assess the tRF‐31‐79MP9P9NH57SD serum expression from NSCLC patients and to determine its diagnostic usefulness.

**Methods:**

By using stem‐loop quantitative real‐time PCR, we were able to detect various tRF‐31‐79MP9P9NH57SD expressions in 96 NSCLC serum samples, 96 healthy controls, and 20 pairs of NSCLC serum samples pre‐ and post‐surgery (qRT‐PCR). After that, we analyzed its diagnostic effectiveness using the receiver operating characteristic (ROC) curve.

**Results:**

Serum tRF‐31‐79MP9P9NH57SD expression was higher in NSCLC patients, and levels of tRF‐31‐79MP9P9NH57SD were linked to the clinical stage (*p* = 0.002) and the malignancy of lymph node (*p* = 0.012). In addition, after the procedure, the serum tRF‐31‐79MP9P9NH57SD expression in NSCLC patients dropped. With 48.96 percent sensitivity and 90.62 percent specificity, the area under ROC curve (AUC) was 0.733.

**Conclusion:**

serum tRF‐31‐79MP9P9NH57SD possibly is a new and groundbreaking biomarker for the NSCLC.

## Graphical abstract

Serum levels of tRF‐31‐79MP9P9NH57SD were detected in NSCLC by stem‐loop qRT‐PCR. Then clinical implications and downstream targets of tRF‐31‐79MP9P9NH57SD were analyzed.

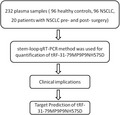

## 1 INTRODUCTION

The most frequent malignant tumor in humans is lung cancer. In China, mortality of lung cancer continually occupies the first place among all malignancies. Non‐small cell lung cancer (NSCLC) is responsible for almost 80 percent of all cases related to lung cancer.^1^ Despite the development of molecular targeted therapies in lung cancers, patients' 5‐year survival estimates are still dismal, mainly because most patients reached an advanced stage when diagnosed.^2^, ^3^ Traditional tumor markers, such as neuron‐specific enolase (NSE) and cytokeratin fragment 21–1 (CYFRA21‐1), were restricted in clinical utilization due to inferior sensitivity and specificity.^4^, ^5^Therefore, it is critical to explore novel and effective biomarkers in NSCLC.

Small non‐coding RNAs (sncRNAs) formed from matured tRNAs or pre‐tRNAs are known as transfer RNA (tRNA)‐derived small RNAs (tsRNAs).^6^ Based on the cleavage site and length, they are divided into two categories: tRNA‐derived fragments (tRFs) and tRNA halves (tiRNAs). tRFs are further split into tRF‐1, tRF‐3, and tRF‐5 based on the tRNA splicing site, whereas tiRNAs are separated into two subtypes based on the anticodon cleavage site: 5’tiRNAs and 3’tiRNAs.^7^, ^8^

Growing evidence suggests that tsRNAs play important roles in multifarious human illnesses due to their part in cancer cells growth, translation regulation, and genetic silencing.^9^, ^10^, ^11^ For example, tRF‐3027 (tRNA^Gly‐GCC^) participates in the development of RNA‐induced silencing complex and silences the replication protein RPA1 to inhibit tumor cell proliferation.^12^ tRF‐5026a (tRF‐18‐79MP9P04) expression was dramatically decreased in the tissues of gastric cancer, according to Zhu et al., and tRF‐5026a could modulate the cancerous cell growth and pathological assault via the signaling pathway of PTEN/PI3K/AKT.^13^ More crucially, some research has looked into the idea of using tRFs as new biological markers for different diseases.^14^, ^15^, ^16^

According to tRFdb^17^ and related databases,^18^ tRF‐31‐79MP9P9NH57SD may be associated with lung cancer. Its diagnostic efficacy and biological involvement in lung cancer, however, are unknown. Here, we investigated the tRF‐31‐79MP9P9NH57SD serum expression in patients suffering from lung cancer. Then, the multifactorial connections of clinical and pathological characteristics were assessed. Our data indicate that tRF‐31‐79MP9P9NH57SD may be used as a noninvasive indicator for diagnosing lung cancer.

## 2 MATERIALS AND METHODS

### 2.1 Patients and samples

Serum from 96 NSCLC patients before surgery, 20 patients with NSCLC pre‐ and post‐ surgery, and 96 healthy human subjects were acquired from 2020 to 2022 at the Ningbo University's affiliated People's Hospital. Before usage, the samples were kept at −80°C. NSCLC diagnosis was confirmed via pathological examination in appropriate patients. Chemotherapy and radiography were not used on the participants who took part in this trial. The present research was performed with the approval of the Ningbo University's affiliated People's Hospital and their Ethical Committee. Informed consent was obtained from all the recruited human subjects.

### 2.2 RNA preparation

TRIzol™ LS Reagent (Invitrogen) was utilized to extract the entire RNA from serum as per the manufacturer's directions. For isopropanol precipitation, glycogen 100 μg/ml (Thermo Fisher) was added, to boost the precipitation of RNA.

### 2.3 qRT‐PCR

To reverse transcribe total RNA, the ImProm‐II Reverse Transcription System (Promega,) was utilized according to the directions provided by the manufacturer. Based on the reported literatures,^14^, ^19^ stem‐loop qRT‐PCR method was used for quantification of mature tRFs, and miR‐16 was selected as an internal control for the quantification of tRFs in serum. The 2‐^−∆∆Ct^ technique was utilized in order to establish the related expression levels. RT and qPCR primers were used in this study: tRF‐31‐79MP9P9NH57SD stem‐loop primer: 5'‐GTCGTATCCAGTGCAGGGTCCGAGGTATTCGCACTGGATACGACGCGAACGT‐3'. The underlined nucleotides are complemented with tRF‐31‐79MP9P9NH57SD. miR‐16 stem‐loop primer: 5'‐ GTCGTATCCAGTGCAGGGTCCGAGGTATTCGCACTGG ATACGACCGCCAATA −3'. The underlined nucleotides are complemented with miR‐16. Forward qPCR primer for tRF‐31‐79MP9P9NH57SD: 5'‐GACGACGTTTCCGTAGTG TAGTG‐3', Forward qPCR primer for miR‐16: 5'‐GTCGCCGTAGCAGCA CGTAAA‐3', Universal Reverse qPCR Primer:5'‐ CCAGTGCAGGGTCC GAGGTA −3'.

### 2.4 Statistical analysis

GraphPad Prism 7.0 (GraphPad, Inc.,) was used for statistical analysis. For data comparison, statistical techniques such as Student's t‐test and chi‐square test were utilized when needed. The ROC curve studies were performed using MedCalc 11.0 (MedCalc, Ostend, Belgium). In these studies, the significance was considered to be at *p* < 0.05.

## 3 RESULTS

### 3.1 Characteristics of the tRF‐31‐79MP9P9NH57SD

**FIGURE 1:**
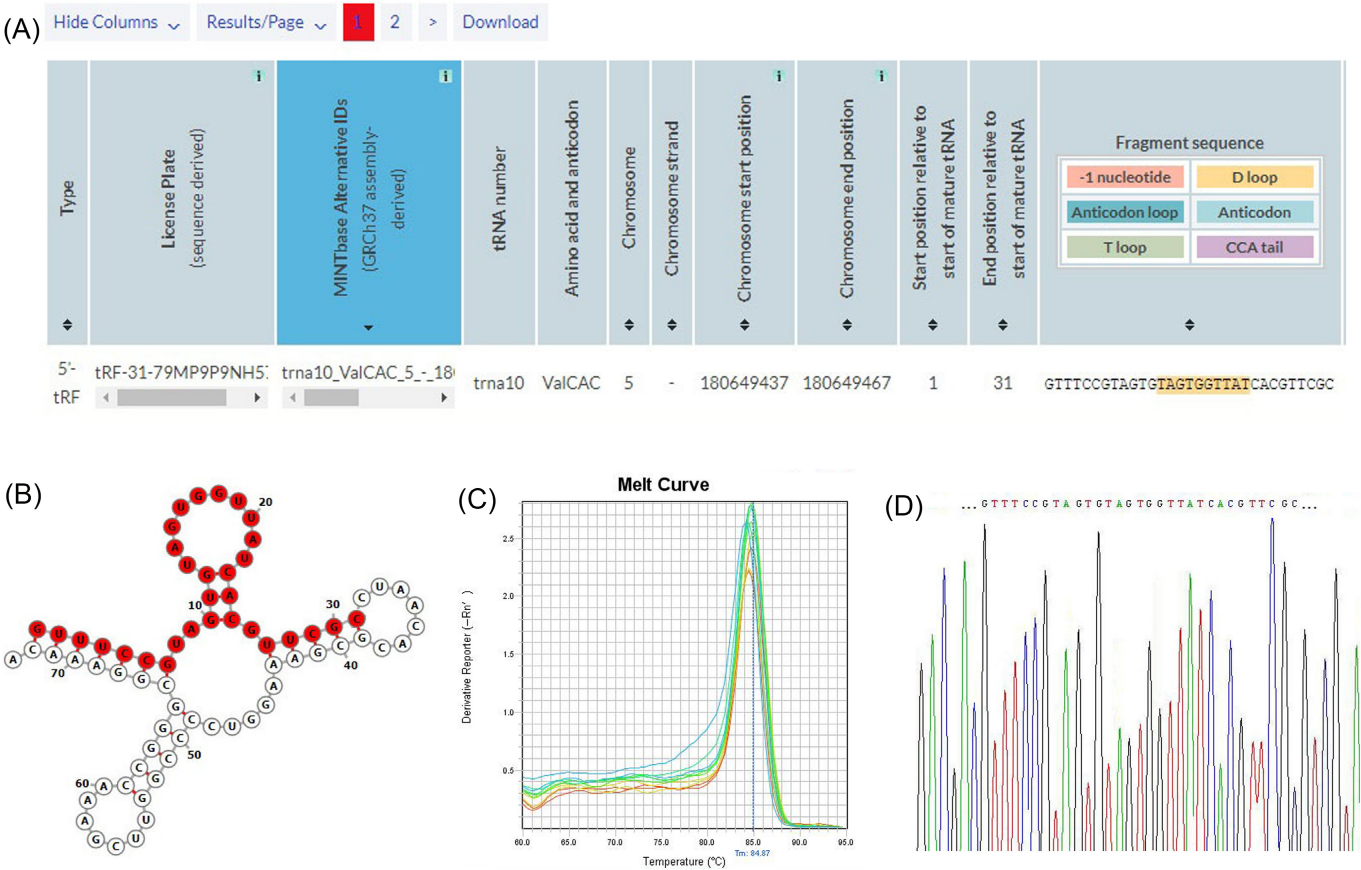
Characteristics of the tRF‐31‐79MP9P9NH57SD. (A) tRF‐31‐79MP9P9NH57SD is a form of 5’‐tRF with a length of 31 nt. (B) tRF‐31‐79MP9P9NH57SD is derived from mature tRNAVal‐CAC. (C) The PCR melting curve of tRF‐31‐79MP9P9NH57SD. (D) Sanger sequencing was used to validate the qRT‐PCR product

In MINTbase v2.0, tRF‐31‐79MP9P9NH57SD is a type of 5’‐tRF with a length of 31 nt (5’‐GTTTCCGTAGTGTAGTGGTTATCACGTTCGC‐3') (Figure 1A). tRF‐31‐79MP9P9NH57SD is derived from mature tRNA^Val‐CAC^, and its secondary structure is shown in Figure 1B. Then, to amplify the tRF‐31‐79MP9P9NH57SD, we created one set of primers, and the amplified products were analyzed through melting curve and Sanger sequencing. Our findings revealed that the amplified product only produced a single peak. (Figure 1C), and the sequences were coincident with that in MINTbase v2.0 (Figure 1D). The data showed that tRF‐31‐79MP9P9NH57SD could be amplified by qRT‐PCR availably.

### 3.2 Highly expressed tRF‐31‐79MP9P9NH57SD in serum of NSCLC patients

**FIGURE 2:**
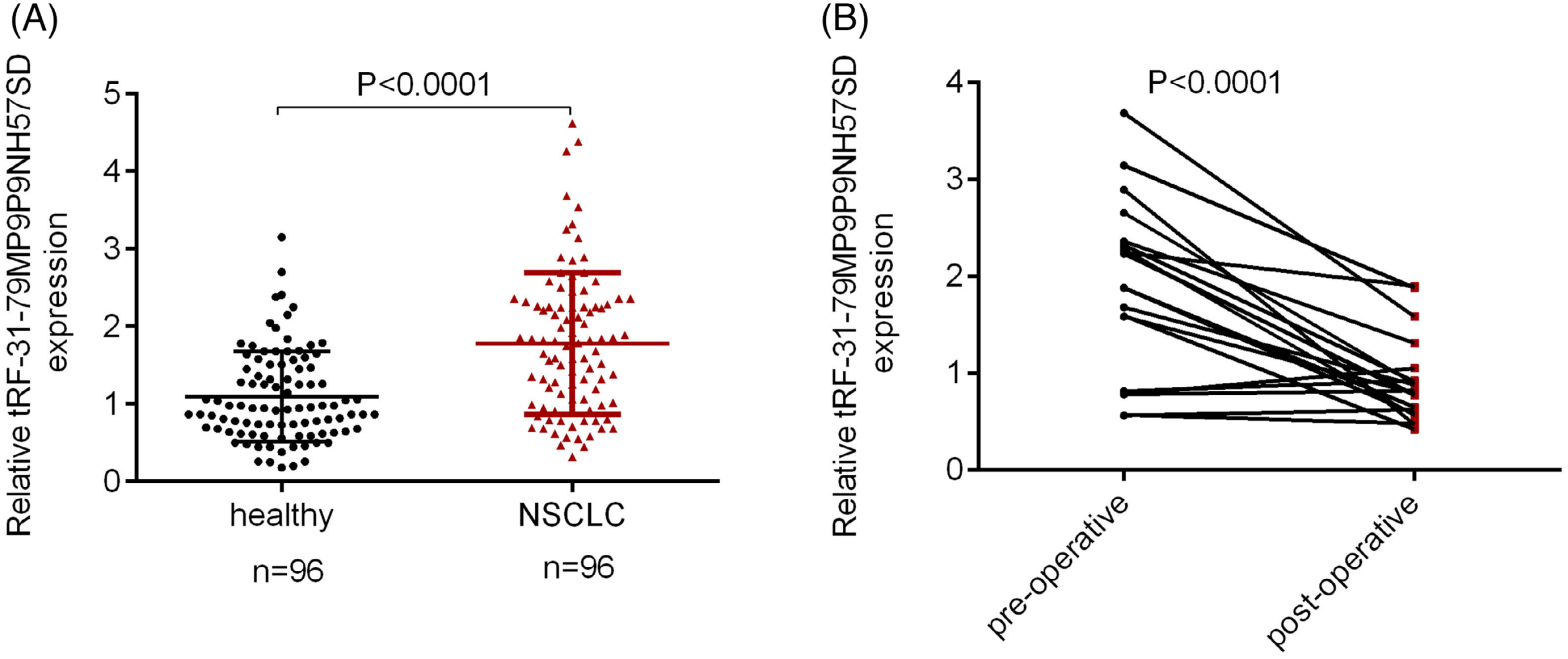
Highly expressed tRF‐31‐79MP9P9NH57SD in serum of NSCLC patients. (A) Stem‐loop qRT‐PCR detected a comparison of tRF‐31‐79MP9P9NH57SD expression between NSCLC patients and healthy human subjects. (B) The tRF‐31‐79MP9P9NH57SD amount in paired pre‐ and post‐surgical samples

qRT‐PCR detected the tRF‐31‐79MP9P9NH57SD expression in serum samples of the 96 NSCLC patients and healthy controls. The expression of tRF‐31‐79MP9P9NH57SD was substantially increased in NSCLC patients as to that of the healthy controls, according to our findings (Figure 2A). Next, tRF‐31‐79MP9P9NH57SD expression in another group including 20 NSCLC pre‐ and post‐surgical patients and 20 healthy human subjects (termed as controls) was detected. The resulting outcome reveals that tRF‐31‐79MP9P9NH57SD was enriched in NSCLC patient's serum before the surgical procedure (Figure 2B).

### 3.3 Clinical implications of serum tRF‐31‐79MP9P9NH57SD in NSCLC

**FIGURE 3:**
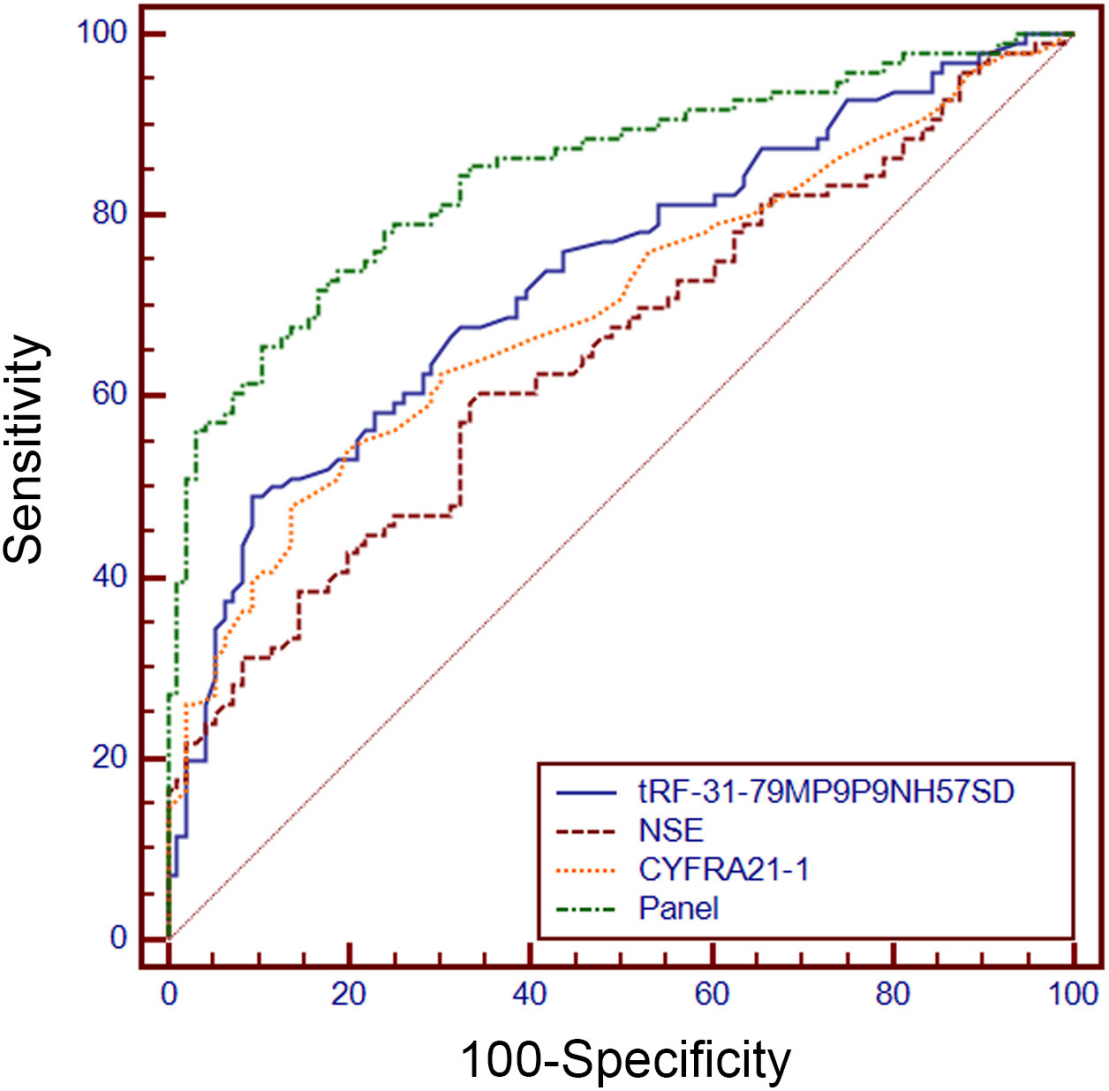
ROC curve assessed the diagnostic value of serum tRF‐31‐79MP9P9NH57SD, NSE, CYFRA21‐1, and the combination of these biomarkers (Panel) in NSCLC

We further explored the connection between the serum tRF‐31‐79MP9P9NH57SD expression and clinicopathological parameters in NSCLC. Based on qRT‐PCR results, the patients were separated into two groups: those with elevated tRF‐31‐79MP9P9NH57SD expression and those with reduced tRF‐31‐79MP9P9NH57SD expression. Table 1 shows a correlation between serum tRF‐31‐79MP9P9NH57SD expression and clinical stage (*p* = 0.002) and lymph node metastases (*p* = 0.012). However, no relationship was detected between tRF‐31‐79MP9P9NH57SD expression and other clinical features, such as age, gender, smoking status, and tumor differentiation stages. Then, the ROC curve was constructed to estimate the diagnostic value of tRF‐31‐79MP9P9NH57SD, NSE, CYFRA21‐1, or a combination of these biomarkers (Panel). The area under the ROC curve (AUC) of tRF‐31‐79MP9P9NH57SD got to 0.733, with 48.96 percent sensitivity and 90.62 percent specificity, as shown in Figure 3. Moreover, we also found that the diagnostic accuracy of the Panel was higher than that of either single biomarker (Table S1 **)**. Collectively, these results suggested that serum tRF‐31‐79MP9P9NH57SD might have potential values for NSCLC.

**TABLE 1:** Association between tRF‐31‐79MP9P9NH57SD expression and clinicopathological characteristics. ^*^ *p* < 0.05

Variable	Number of cases	tRF‐31‐79MP9P9NH57SD expression		*p value*
		High expression (*N* = 48)	Low expression (*N* = 48)	
Age				0.151
≥60	52	30	22	
<60	44	18	26	
Gender				0.203
Female	35	21	14	
Male	61	27	34	
Smoking status				1.000
Yes	55	28	27	
No	41	20	21	
Differentiation grade				0.307
Well‐moderate	50	22	28	
Poor‐undifferentiation	46	26	20	
Histological subtype				0.516
Squamous cell carcinoma	32	18	14	
Adenocarcinoma	64	30	34	
Clinical stage				0.002
I + II	72	29	43	
III + IV	24	19	5	
Lymph node metastasis				0.012
Negative	80	35	45	
Positive	16	13	3	
Tumor size (cm)				0.324
<5	75	35	40	
≥5	21	13	8	

### 3.4 Exploration of the downstream and function prediction of tRF‐31‐79MP9P9NH57SD

**FIGURE 4:**
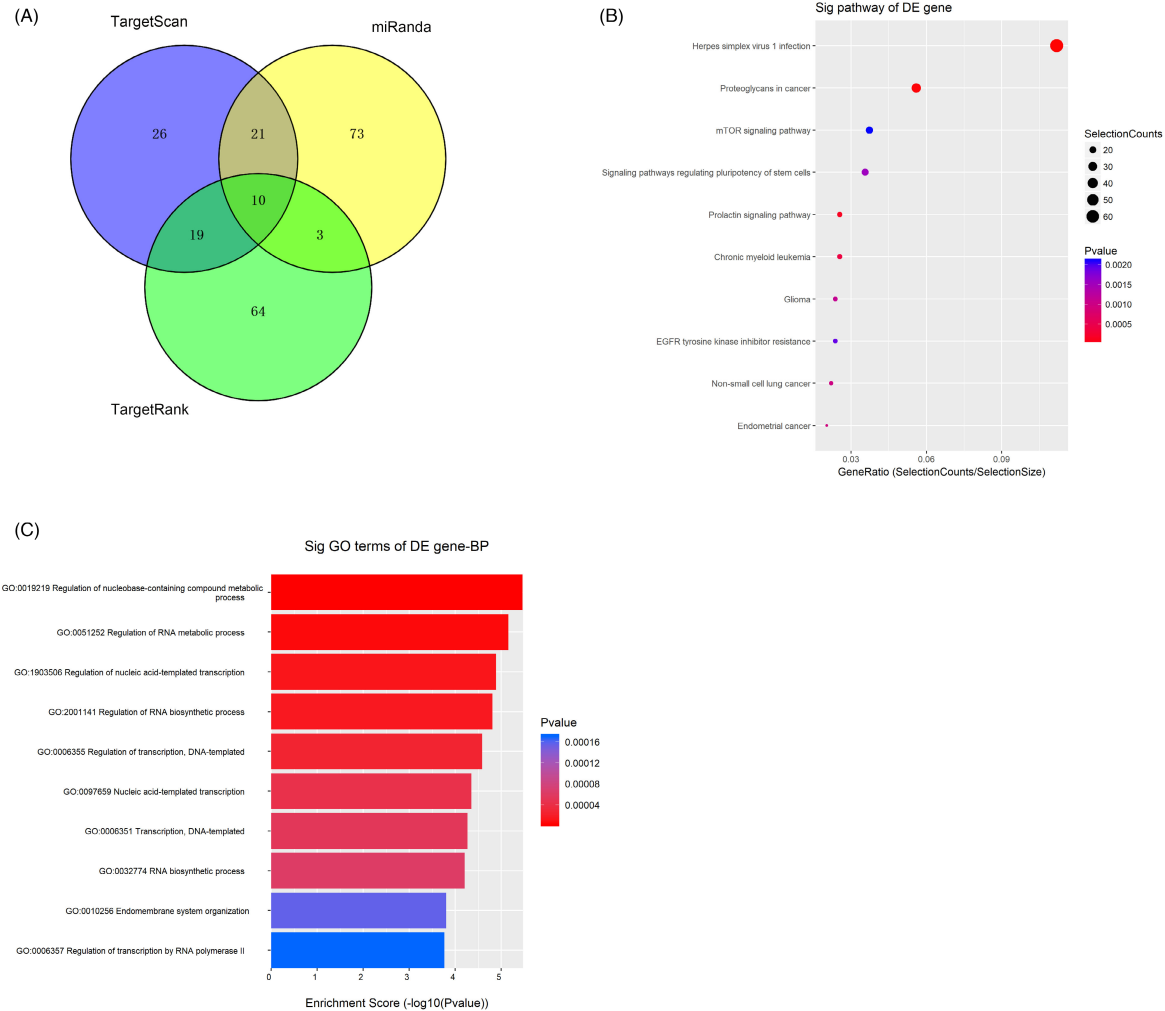
Investigation of the downstream and role estimation of tRF‐31‐79MP9P9NH57SD. (A) Possible targeted genes of tRF‐31‐79MP9P9NH57SD as anticipated by TargetScan, miRanda, and TargetRank. (B) KEGG pathway analysis of tRF‐31‐79MP9P9NH57SD. (C) GO enrichment analysis of tRF‐31‐79MP9P9NH57SD

In the TargetScan, miRanda, and TargetRank databases, we predicted downstream targets of tRF‐31‐79MP9P9NH57SD in a Venny diagram (Figure 4A). Among the linked genes that demonstrated the most overlap were ten genes (SACS, ERGIC2, KIF5C, IGF1R, ATP10D, MAP3K2, GALNT10, RMND5A, ZNF33B and OAS3). We then sought to look into the molecular mechanism of tRF‐31‐79MP9P9NH57SD. KEGG Enrichment analysis indicated that tRF‐31‐79MP9P9NH57SD was enriched in virus infection, a variety of tumors and mTOR signaling pathway (Figure 4B). tRF‐31‐79MP9P9NH57SD may have a role in RNA biosynthesis and transcription control, according to GO functional enrichment analysis of the target genes (Figure 4C). Therefore, the mechanisms of tRF‐31‐79MP9P9NH57SD in the regulation of NSCLC need to be further investigated.

## 4 DISCUSSION

The primary function of tRNAs is to transport amino acids to the ribosome, allowing the synthesis of the appropriate protein to proceed more quickly under the supervision of the mRNA. tRFs and tiRNAs have lately been discovered in a range of malignancies,^20^ according to some research. By regulating transcription, modifying mRNA stability, silencing target genes, and engaging in the cellular stress response, tRFs and tiRNAs can influence cancer formation.^21^, ^22^, ^23^ Han et al. reported that tRF‐3008a suppresses colorectal cancer (CRC) metastasis by repressing endogenous FOXK1, and it could be utilized as a possible prognostic biological marker for CRC.^24^ Parallel outcomes were observed that tRF‐3008a could suppress cells malignant activity silencing THBS1 in breast cancer.^25^

Recently, several tRFs have been reported to be diagnostic biomarkers in lung cancer and other malignancies.^15^, ^26^, ^27^ In this study, we screened tRFs from high‐throughput sequencing databases and found tRF‐31‐79MP9P9NH57SD may be a lung cancer‐associated tRF. Then, stem‐loop qRT‐PCR method was used for quantification of tRF‐31‐79MP9P9NH57SD. Our results showed that the NSCLC patients exhibited dramatically increased serum quantities of tRF‐31‐79MP9P9NH57SD compared with healthy donors. Statistical analysis demonstrated that increased tRF‐31‐79MP9P9NH57SD expression was directly linked to tumor size (*p* = 0.001) and malignant condition of the lymph node (*p* = 0.038). After measuring serum tRF‐31‐79MP9P9NH57SD expression pre‐ and post‐surgical patients, it was discovered that levels of tRF‐31‐79MP9P9NH57SD declined dramatically after surgery, which could be a symptom of tumor recurrence. Zhu et al. reported that tRFs could be secreted from tumor cells by exosomes.^14^ We speculate that tRF‐31‐79MP9P9NH57SD might be secreted into serum by exosomes. However, this still needs further experiments to confirm.

At present, the mechanisms underlying tRFs on cancer occurrence are largely unknown. Bioinformatics analysis employing mRNA target‐predicting techniques was used to investigate the downstream targets of tRF‐31‐79MP9P9NH57SD.^28^, ^29^ Ten linked genes exhibited the most overlap between these approaches. Next, we need to carry out corresponding experiments to identify the targets and functions of tRF‐31‐79MP9P9NH57SD in NSCLC.

## 5 CONCLUSION

To conclude, this research discovered that serum tRF‐31‐79MP9P9NH57SD expression is elevated in NSCLC, and has strong diagnostic importance, insinuating that tRF‐31‐79MP9P9NH57SD serum may be a novel diagnostic marker for NSCLC.

## 6 AUTHOR CONTRIBUTIONS

The study was conceived and carried out by LJ and YW. CC and FL were involved in obtaining ethical approval, collecting samples, performing qRT‐PCR, and analyzing data. The manuscript's first version was written by LJ. The final manuscript was reviewed and approved by all authors.

## 8 CONFLICT OF INTERESTS

None.

9

## 10 CONSENT FOR PUBLICATION

Not applicable.

## Supporting information


Table S1

## Data Availability

On reasonable request, the corresponding author will provide the datasets used and/or analyzed during the current work.
