# Supplementary material for: Serum transfer RNA‐derived fragment tRF‐31‐79MP9P9NH57SD acts as a novel diagnostic biomarker for non‐small cell lung cancer
Source: J Clin Lab Anal. 2022 May 16;36(7):10.1002/jcla.24492. doi: 10.1002/jcla.24492 (PMC9279995; doi:10.1002/jcla.24492)
Supplement: Supplementary file 1 — Table S1 [file JCLA-36--s001.docx]

Table S1. The performance of serum tRF-31-79MP9P9NH57SD, NSE, CYFRA21-1 and their combination for the diagnosis of NSCLC

| Groups | AUC (95% CI) | Sensitivity (%) | Specificity (%) | Comparation of AUC | |
| --- | --- | --- | --- | --- | --- |
|  |  |  |  | Groups | *P value* |
| tRF-31-79MP9P9NH57SD | 0.733(0.665-0.794) | 48.96 | 90.62 | tRF-31-79MP9P9NH57SD vs NSE | 0.110 |
| NSE | 0.650(0.578-0.717) | 60.42 | 65.62 | tRF-31-79MP9P9NH57SD vs CYFRA21-1 | 0.514 |
| CYFRA21-1 | 0.699(0629-0.763) | 47.92 | 86.46 | CYFRA21-1 vs NSE | 0.367 |
| Panel | 0.847(0.788-0.895) | 73.96 | 81.25 | Panel vs tRF-31-79MP9P9NH57SD | 0.0001 |
|  |  |  |  | Panel vs NSE | < 0.0001 |
|  |  |  |  | Panel vs CYFRA21-1 | < 0.0001 |
